# SeqSNP-based genic markers reveal genetic architecture and candidate genes for low nitrogen tolerance in tropical maize inbred lines

**DOI:** 10.3389/fpls.2025.1558741

**Published:** 2025-05-30

**Authors:** Pearl Abu, Baffour Badu-Apraku, Beatrice Elohor Ifie, John Saviour Yaw Eleblu, Georgina Lala Ehemba, Pangirayi B. Tongoona, Samuel Kwame Offei

**Affiliations:** ^1^ West Africa Centre for Crop Improvement, University of Ghana, Accra, Ghana; ^2^ International Institute of Tropical Agriculture (IITA), Ibadan, Nigeria; ^3^ Institute of Biological, Environmental and Rural Sciences (IBERS), Aberystwyth University, Aberystwyth, United Kingdom

**Keywords:** maize, low soil nitrogen, high soil nitrogen, gene targeting markers, population structure, candidate genes, SeqSNPs

## Abstract

Maize production in sub-Saharan Africa (SSA) faces significant challenges due to low soil nitrogen. To enhance breeding efficiency for low nitrogen tolerance, identifying quantitative trait loci (QTLs) in tropical germplasm is crucial to facilitate marker-assisted selection (MAS). In this study, gene targeting markers (GTM) derived from sequence-based single nucleotide polymorphisms (SeqSNP) were utilized to analyse the population structure and identify potential candidate genes associated with tolerance to low nitrogen. A total of 150 extra-early quality protein maize (QPM) inbred lines were assessed under both low (LN) and high (HN) nitrogen, followed by genotyping with 2,500 SeqSNPs targeting genes previously reported for LN tolerance-related traits. Population structure analysis revealed six sub-populations. Association mapping analysis revealed 15 significant single nucleotide polymorphisms (SNPs) linked to several key traits. Specifically, two SNPs each were associated with the low nitrogen base index (LNBI), which combines grain yield with other agronomic traits under low nitrogen, and the low nitrogen tolerance index (LNTI), a measure of grain yield performance in high nitrogen environments relative to low nitrogen environments. Additionally, one and ten SNPs were identified for grain yield under low and high nitrogen conditions, respectively. The two SNPs associated with LNTI were found to co-localize a potential gene hotspot, GRMZM2G077863, which belongs to the GDSL esterase/lipase gene family and is highly expressed in the roots of young seedlings six days after planting and during tassel meiosis prior to flowering. Additionally, several other putative genes were identified across different chromosomes: GRMZM2G026137 and GRMZM2G004459 on chromosome 1, GRMZM2G111809 on chromosome 2, GRMZM2G380319 on chromosome 3, GRMZM2G442057 and GRMZM2G080314 on chromosome 6, GRMZM2G011213 and GRMZM2G090928 on chromosome 8, and GRMZM2G338056 and GRMZM2G150598 on chromosome 9. The genes are involved in several functions including normal growth, tassel meiosis, root architecture, cell proliferation, cell growth, reproduction, and post-embryonic development. We report PZE-103012466, a marker co-localizing GRMZM2G380319, which was previously found to be associated with root elongation, as a useful marker for breeding low soil nitrogen tolerance in tropical germplasm. The validation of these markers and candidate genes in other populations could make them useful for MAS in breeding for nitrogen tolerance.

## 1 Introduction

Nitrogen (N) is an essential soil nutrient required in a relatively high supply to support several critical growth and developmental functions in cereal crops (Qiu et al., 2015; Morosini et al., 2017). Nitrogen is a major component of nucleic acids and is involved in several functional processes such as protein synthesis, photosynthesis, and carbohydrate production (Bänziger et al., 2000). In maize, nitrogen deficiency causes retarded leaf development and accelerated leaf senescence, resulting in lower leaf area indices, leading to decreased photosynthetic capacity and reduced grain yield (Bänziger et al., 2000; Asibi et al., 2019). Low soil nitrogen (LN) related yield losses range between 10-50% but can reach 90%, especially when it co-occurs with other stresses such as drought (Obeng-Bio et al., 2019).

Despite the significant relationship between N and maize productivity, N is highly limited in most soils globally. Africa has the most severe case of LN-related yield loss of 80%, largely because of the intrinsically marginal soil nutrient conditions and inadequate investment by governments in addressing abiotic stress constraints (Bänziger et al., 2006). This problem is further aggravated by farmers’ inability to afford soil replenishment interventions such as organic and inorganic fertilizers (Morosini et al., 2017). Thus, farmers continue to cultivate maize at suboptimal nitrogen levels below the 90–120 kg ha^-1^ recommended rate (Das et al., 2019), resulting in low yields. The development of LN-tolerant varieties that can produce substantial yields under the prevailing N conditions in smallholder farmers’ fields in sub-Saharan Africa (SSA) is deemed the most appropriate approach, as it is cost-effective, sustainable and easily integrated into farming systems.

Breeding for LN tolerance is complex because the trait is controlled by multiple genes (Gallais and Hirel, 2004; Morosini et al., 2017), highly influenced by genotype × environment (G × E) interaction effects, and characterized by lower grain yield heritability estimates (Bänziger et al., 2000; Badu-Apraku et al., 2004). Furthermore, grain yield, which represents the economically significant trait in breeding for LN tolerance, is moderated by many quantitative trait loci (QTLs), making per *se* performance-based selection under field conditions less efficient. Therefore, many breeding programmes have employed secondary traits such as stay-green characteristics, number of ears per plant, anthesis–silking interval (ASI) and ear aspect due to their relatively higher heritability and strong correlation with grain yield under abiotic stress (Bänziger et al., 2000; Badu-Apraku et al., 2011). Nonetheless, most of these traits are only measurable post-flowering and harvest, limiting their usefulness in breeding programmes especially for pre-flowering selection, to enable targeted crossing of desirable genotypes.

Molecular markers have been useful for dissecting the genetic structure of populations, studying the genetic relationship between related and distant populations and enabled the application of genomic selection and association mapping studies to accelerate the breeding of complex traits such as grain yield. Marker-assisted selection (MAS) offers an accelerated and effective selection strategy for crop improvement programmes in breeding for biotic and abiotic stresses. Marker-assisted breeding increases selection efficiency through reduced environmental influence, and saves cost due to the possibility of integrating pre-flowering data to enable controlled crosses (Massman et al., 2013). However, the efficiency of MAS for the introgression of relevant genomic regions into available tropical populations depends on the availability of markers with significant association to QTLs related to the target trait and their reliability in specific genetic backgrounds.

Genome-wide association studies (GWAS) employed in various research to identify genomic regions and candidate genes have improved the efficiency in selection for biotic and abiotic traits in maize breeding programmes. Many researchers have integrated GWAS to identify QTLs for days to flowering (Wallace et al., 2016), aflatoxin resistance (Farfan et al., 2015), *Striga* resistance (Adewale et al., 2020), drought tolerance (Yuan et al., 2019), provitamin A content (Azmach et al., 2013), and heat stress (Longmei et al., 2021). Several QTLs have also been reported for LN stress-related traits. For instance, Morosini et al. (2017) identified seven significant markers for nitrogen use efficiency (NUE) in sixty-four inbred lines of tropical ancestry and reported seven putative candidate genes involved in biological processes related to nitrogen synthesis and recycling. Bhadmus et al. (2022), in a study with tropical quality protein maize (QPM) inbred lines developed under the maize improvement programme (MIP) of the International Institute of Tropical Agriculture (IITA), reported 40 SNP markers that were significantly associated with NUE. The authors also identified putative candidate genes that contributed significantly to nitrogen uptake and biosynthesis, normal plant growth and development, and disease resistance. Ertiro et al. (2020) found 83 SNP that were associated with yield related traits under LN and optimal environments in tropical inbred lines from the International Maize and Wheat Improvement Center (CIMMYT). The authors reported pleiotropic effects of some SNPs for several traits and identified 136 putative candidate genes under LN and optimal growing conditions, four of which are involved in important biological functions related to shorter ASI. Other studies have also mapped QTLs for LN tolerance by using linkage mapping analysis (Cai et al., 2012; Mandolino et al., 2018; Ribeiro et al., 2018).

Even though several QTLs for LN tolerance have been identified in different populations with varying genetic backgrounds, their relevance for MAS is often limited to specific populations and agro-ecologies and, hence may not be applicable to other germplasm (Ribeiro et al., 2018). Therefore, to effectively integrate or introgress the reported QTLs for accelerated breeding of LN tolerant varieties through MAS, their availability and significance in our tropical QPM maize germplasm is necessary. Currently, under the MIP of the IITA, we have a collection of 160 QPM inbred lines that are useful genetic resources that could be exploited in breeding for LN tolerance. However, there are no available markers to enable the integration of MAS, hence, it is crucial to identify molecular markers and candidate genes that are associated with nitrogen use efficiency (NUE) to optimize selection for genetic gains in our population which can also be applied in other tropical backgrounds.

Gene targeting markers (GTMs) are a class of molecular markers that targets specific genes in the genome (Poczai et al., 2013). When GTMs are positioned inside coding regions for a target gene of interest, they are more informative compared to non-genic markers which rely solely on their expected associations with the target genes or loci (Osterman et al., 2021). By designing SNPs that are located within coding sequences, it is expected that the probability of accurately describing the genetic diversity within and between population with respect to a specific trait of interest would increase (van Tienderen et al., 2002).

SeqSNP is a refined form of targeted genotype by sequencing (targeted GBS) technique used to genotype known highly polymorphic SNPs in a target crop. Unlike whole-genome sequencing via the GBS technique, SeqSNP, which is a GTM technique, relies on a probe library design that surrounds a targeted SNP sequence. This offers flexibility in SNP sequence selection, ultimately allowing breeding programmes to focus on the genomic regions that are relevant to the target trait. In circumstances where desirable SNPs for a specific trait are not available, *de novo* SNPs candidate gene identification can be achieved via allele mining of available genomic resources of the crop of interest (Osterman et al., 2021). Subsequently, the importance of these novel SNPs for a particular genetic resource can be tested through population structure, based on which a core set of SNPs could be identified for improving the genetic resource through MAS. The availability of publicly accessible sequence data for maize in the maize genome database (Andorf et al., 2010) enabled the application of the SeqSNP for our studies. At the time of this study, the latest genome was the B73 reference genome assembly (Schnable et al., 2009) version 3; the annotations and assemblies enabled the determination of gene sequences and SNP markers targeting LN tolerance related traits in our inbred lines. Using gene-targeting SNPs located within coding sequences for association mapping studies, together with phenotypic information on LN tolerance, markers with strong association to the traits could be identified in our tropical QPM population for efficient MAS and could be applied in other tropical populations. Therefore, the aim of this research was to determine SNP markers and candidate genes that are significantly linked to LN tolerance and yield-related traits in the tropical QPM inbred lines.

## 2 Materials and methods

### 2.1 Genetic material

This study used 160 extra-early QPM inbred lines obtained from the maize improvement programme (MIP) of the International Institute of Tropical Agriculture (IITA). They consisted of 53 yellow, 57 orange, and 50 white QPM inbred lines. These selected lines were considered to be diverse enough for the marker traits association study because they were extracted from three different stress tolerant populations and are of different endosperm colours.

### 2.2 Phenotyping under low and high soil nitrogen

The lines were screened under low (LN) and high (HN) nitrogen field conditions during the major and minor growing periods of 2019 in Ghana. The trials were conducted in three locations; the Research Farm of the West Africa Centre for Crop Improvement (WACCI), University of Ghana Legon (5° 38–45 N lat., 00° 11–13 E long.) during the minor growing season, the Crop Research Institute (CRI)- Fumesua (6°41′ N lat., 1°28′ W long.) during both the major and minor growing seasons, and at Ejura (7^0^ 40N and1^0^ 39W) during the minor growing season. Location-by-season combinations were considered as environment, which resulted in four (Legon, Fumesua-major season, Fumesua-minor season and Ejura-minor season) and three (Fumesua-major season, Fumesua-minor season and Ejura-minor season) environments for the LN and HN experiments, respectively. The soils in all experimental fields were planted with maize at a high population density for at least one growing season to deplete N and achieve N levels ≤ 0.2% (Landon, 1991). After the depletion, soil samples were taken for laboratory analysis following the procedure described by Bremner and Mulvaney (1982). The soil analysis revealed low nitrogen levels of 0.04%, 0.05% and 0.16% at the Ejura, Legon and Fumesua experimental sites respectively.

The experiment was arranged in a 10 × 16 alpha lattice design and was replicated twice. A 4 m long plot size having 0.4 m intra-row spacing and 0.75 m inter-row spacing was adopted. Initially, three seeds were planted in each hole but was reduced to two plants at two weeks after planting to achieve 66,666 plants per hectare for the experiment. The LN trials were supplied with a quantity of fertilizer (urea) based on the levels reported in the soil analysis to bring the level to 30 kg N ha^-1^ while the HN trials were fertilized with 90 kg N ha^-1^. In both the HN and LN trials, nitrogen fertilizer was applied when the plants were two and five weeks old. All experiments received phosphorus as triple super phosphate and potassium applied as muriate of potash at 60 kg ha^-1^ each.

### 2.3 Phenotypic data collection

Data were collected as described by Badu-Apraku et al. (2016). Briefly, days to anthesis (DA) were recorded as the number of days from planting to when 50% of the plants in a plot had shed pollen. Days to silking (DS) were obtained as the number of days from planting to when 50% of the plants in a plot produced silk. The anthesis silking interval (ASI) was estimated as the difference between DS and DA. Stay-green characteristics (STGR) and plant aspect (PA) were scored 70 days after planting (DAP). The STGR was scored based on leaf senescence using a scale of 1–9, where 1 = 0–10% dead leaf area and 9 = 91–100% dead leaf area. PA was scored on a scale of 1–9, where 1 = excellent plant phenotypic appearance and 9 = poor plant phenotypic appearance. The number of ears per plant (EPP) was calculated by dividing the number of ears harvested per plot by the number of plants harvested. The ear aspect (EA) was scored at harvest, after removing the husk, on a scale of 1–9, where 1 = uniform, well-filled, large, and disease-free ears, and 9 = undesirable ear features. Grain yield (kg ha^-1^) in each plot was calculated using the grain weight (kg), the shelled weight of harvested ears per plot, and moisture content of the shelled ears and 15% adjusted moisture content. Two methods were used to determine LN tolerance in this study. The first method employed the low nitrogen base index (LNBI) according to Badu-Apraku et al. (2011) as indicated below;


LNBI=2×GYLD+EPP+ASI+PA+EA+STGR

Where GYLD is grain weight, EPP is number of ears per plant, ASI is anthesis-silking interval, PA is plant aspect, EA is ear aspect and STGR is stay-green characteristics. Under this index, the higher the LNBI, the greater is the tolerance of the line to LN stress. The second method employed a low nitrogen tolerance index (LNTI) proposed by Miti et al. (2010), using grain yield in each replication under HN and LN within the same environment;


$$LNTI=(1-\frac{GY{\left(LN\right)}_{ij}}{GY{\left(HN\right)}_{ij}})\times \;100$$

GY_(LN)ij_ denotes the GY recorded in a LN environment of the i^th^ line in the j^th^ replication; GY(HN)ij denotes the GY of the i^th^ line in a HN environment in the j^th^ replication. In this index, a lower LNTI value indicates greater tolerance to LN stress.

### 2.4 Genotyping of inbred lines

Leaf samples were collected from 150 inbred lines instead of the 160 lines phenotyped for genotyping because ten of the lines were lost in the nursery due to poor germination. The leaf samples were sent to LGC genomics, Germany for genotyping via the targeted-genotyping-by-sequencing (SeqSNP) platform (https://www.biosearchtech.com/services/sequencing/targeted-genotyping-by-sequencing-seqsnp). The SeqSNP technology, which uses gene targeting markers, enabled the selection of important SNPs targeting QTLs and genomic regions relevant to the traits of interest. The markers selected for this study targeted SNPs located within or adjacent to QTL positions originally reported for grain yield and LN tolerance traits, such as STGR, ASI and plant height in QTL mapping studies or GWAS (Almeida et al., 2013; 2014; Nair et al., 2015; Trachsel et al., 2016; Bouchet et al., 2017; Mandolino et al., 2018; Morosini et al., 2017; Ribeiro et al., 2018; Ju et al., 2018) (**Supplementary Table 1**). For QTL studies employing simple sequence repeat (SSR) markers, the physical positions of SSR markers associated with relevant QTLs were obtained from the maize genome database (Lawrence et al., 2004; https://www.maizegdb.org/) to guide the selection of SNPs within or adjacent to these QTLs. A total of 2,500 SNPs were chosen from the 50 K maize SNP genotyping array developed by Ganal et al. (2011). Out of the 2500 markers, 90 SNPs with exact positions, as previously reported for QTLs were found in the 50 K array. To ensure that the relevant QTLs were targeted, 2000 SNPs were selected close to (two markers each upstream and downstream) the previously reported QTL positions because the exact SNP positions were not available in the 50 K array. Additionally, 410 random markers distributed across the ten chromosomes were selected for possible discovery of novel QTLs within the tropical QPM populations used for this study.

### 2.5 Data analysis

#### 2.5.1 Phenotypic data analysis

Analysis of variance was performed for all traits via the multi-environment trait analysis with R for windows (META-R) software (Alvarado et al., 2016). The Restricted Maximum Likelihood/Best Linear Unbiased Predictor (REML/BLUP) was used to estimate the predicted means of the inbred lines for each trait under the LN and HN conditions. For the mixed model, the effects of environments were considered as fixed, while replication within blocks, block within replication, genotype, and genotype × environment were considered as random.

The statistical model underlying the predicted means was;


y=Xe+Ur+Zg+Wb+Ti+ϵ

In this model, *y* denotes the vector of phenotypic values of the inbred lines for the traits under LN and HN, *e* denotes the main effects of environments, *r* represents the effects of replications within environments, *g* denotes the genotype effect, *b* is the effects of blocks within replications, *i* is the effects of genotype × environment interaction, and *ϵ* represents experimental error. *X, U, Z, W* and *T* represent the incident matrices for the independent effect vectors (*g, r, b, e* and *i*) on the dependent vector *y*. Genotypic and phenotypic variance components BLUP estimates were employed to compute the broad sense heritability (H^2^) for all traits;


$${\text{H}}^{2}=\sigma_{\text{g}}^{2}/(\sigma_{\text{g}}^{2}+\sigma_{\text{ge}}^{2}/\text{e}+\sigma_{\text{e}}^{2}/\text{re})$$

*σ^2^_g_* represents the genotypic variance, *σ^2^_ge_* represents the variance due to genotype x environment interaction, *σ^2^_e_* represents the error variance, *e* represents the number of environments and *r* is the number of replicates per environment (Knapp et al., 1985). Furthermore, genetic correlations among traits were estimated using correlation between BLUPs for the different traits.

#### 2.5.2 Genotypic and association mapping data analysis

SNPs having missing data above 10% were not included in the analysis. Additionally, SNPs having minor allele frequency (MAF) below 5% were excluded since markers with low MAF could result in false-positive associations. The filtering was done in TASSEL version 5.2.53 (Bradbury et al., 2007). Only SNPs with call rate > 90% were retained. PowerMarker version 3.25 (Liu and Muse, 2005) was used to estimate polymorphic information content (PIC), heterozygosity, gene diversity and major allele frequency (MaF) for all the markers. Further quality control was done by removing all SNPs with heterozygosity above 20%. After filtering and other quality control procedures, 1,660 markers were retained for further analysis. An unrooted neighbor-joining (NJ) tree was generated using the Nei’s method (Nei and Takezaki, 1983) through 1000 nonparametric bootstraps replicated across several loci. The tree was subsequently viewed in the Molecular Evolutionary Genetics Analysis (MEGA) software X (Kumar et al., 2018) and edited in Figtree software version 1.4.4 (Rambaut, 2018). To determine the population structure, the admixture procedure of STRUCTURE software version 2.3.4 was used. The K, which indicates the number of clusters, was originally set from 1–12 which was run ten times with 10,000 burn-ins and 10,000 Markov Chain Monte Carlo (MCMC). Results from this analysis were fed into STRUCTURE HARVESTER (Evanno et al., 2005) to determine the best K, based on which the inbred lines were grouped into clusters using a threshold of 70% and those that fell below the threshold were grouped as admixture (Lu et al., 2009; Yang et al., 2011). Marker-trait association analysis was performed using the Q+K model with the mixed linear model procedure in TASSEL version 5.2.53 (Bradbury et al., 2007). The principal components (PC1 and PC2), together with the population structure (Q matrix) accounted for population stratification while the kinship matrix (K) accounted for relationships among the lines using the following model;


y=Xβ+Zu+e

*y* represents the vector of phenotypes, *β* represents a vector of the overall mean and the fixed effect estimate of each SNP, *u* is a vector of the additive genetic background effects of a random line, *X* and *Z* denotes incidence matrices and *e* represents a vector of random residuals. The conservative Bonferroni correction factor –log10 (p-value) = 7 was considered too strict for determining significant associations in this study, given that most (83.6%) of the SNPs used were located within or adjacent to QTLs previously identified for the trait of interest. A threshold of –log10 (p-value) = 3 was adopted to avoid losing potentially important genomic regions to a stricter threshold. A similar threshold has been adopted in previous studies (Liu et al., 2016; Alves et al., 2019).

#### 2.5.3 Candidate gene discovery

Candidate genes co-localizing or adjacent to genomic regions for significantly associated SNPs were determined based on the B73 reference genome assembly (Schnable et al., 2009) version 3. The maize genome database (Maize GDB) genome browser tool (Andorf et al., 2010), accessible at https://www.maizegdb.org/gbrowse/maize_v3 was used for candidate gene discovery. Only genes co-localizing with or positioned near significant SNPs within a maximum of one kilo base pair (1kb) sliding window were considered as candidate genes. Transposable elements were not considered in this study. Furthermore, putative functional annotations of the candidate genes were retrieved via Phytozome (Goodstein et al., 2012) using Phytozome 12, version AGPv3 - Zea mays Ensembl-18.

## 3 Results

### 3.1 Phenotypic variation and genetic correlation

In LN environments, significant genotypic variations were detected among the inbred lines for all the measured traits (**Table 1**). The results of the heritability analysis demonstrated moderate to high values for most measured traits in the LN environments with GY having 65%. Genotype × environment (G × E) interaction variances were significant for all measured traits except GY. Considering the HN environments, genetic variations among the inbred lines were significant for all measured traits, however, variances for the G x E suggested significant differences for all the measured traits including GY. The estimated heritability values displayed moderate to high values for majority of the measured traits with heritability of GY estimated as 79%. In the LN environments, STGR ranged between 3.5 – 7.2 while it was 2.5 – 4.8 under HN environments (**Supplementary Table 2**). Genetic correlations among traits were significant for most pairwise traits in LN and HN environments. In the LN environments, GY had a positive genetic correlation with EPP, however, the correlation between GY and ASI, PA and EA were negative and significant (**Table 2**). Although the correlation between GY and ASI, EA and PA were negative, it was desirable in this context because shorter ASI, excellent EA and PA resulted in increased GY. No significant correlation was detected between GY and STGR under LN. Considering the genetic correlation among traits in the HN environments, the correlation between GY and EPP was positive and significant, while GY had a negative and significant correlation with STGR, PA, and EA.

**Table 1:** Estimates of variance components and heritability for traits under low nitrogen (LN) and high Nitrogen (HN) Conditions.

Traits	Variance components				Heritability (H^2^)	Range
	σ^2^p	σ^2^g	σ^2^gxe	σ^2^error		
LN Environment						
ASI	0.865	0.437**	0.792**	1.843	0.5	1.6 - 6.0
STGR 10 WAP (1-9)	0.41	0.277**	0.174**	0.72	0.67	3.5 -7.2
Plant aspect (1-9)	0.236	0.074**	0.307**	0.684	0.31	3.9 - 6.4
Ears per plant	0.017	0.006**	0.028**	0.031	0.37	0.2- 1.0
Ear aspect (1-9)	1.038	0.444**	1.117**	2.514	0.43	3.3 - 7.3
Grain yield (kg ha^-1^)	72093	57206**	9480ns	100142	0.65	151 -1349
High N Environment						
ASI	0.182	0.103**	0.002ns	0.468	0.57	1.3 - 2.8
STGR 10 WAP (1-9)	0.172	0.090**	0.050*	0.392	0.52	2.5 - 4.8
Plant aspect (1-9)	0.237	0.107**	0.095**	0.593	0.45	2.7 - 4.9
Ears per plant	0.037	0.022**	0.014**	0.064	0.59	0.5 - 1.2
Ear aspect (1-9)	1.504	0.594**	1.889**	1.682	0.4	3.2 – 6.1
Grain yield (kg ha^-1^)	133025	86930**	64515**	147540	0.79	258 - 1681

STGR, stay-green characteristics; σ^2p^, phenotypic variance; σ^2g^, genotypic variance, σ^2gxe^, genotype × environment interaction variance; σ^2error^, error variance; *(p ≤ 0.05); **p ≤ 0.01); ns (not significant).

**Table 2:** Genetic correlations coefficients of different traits with grain yield in LN environments.

Traits	ASI	STGR	Plant aspect	Ears per plant	Ear aspect
Low N environment					
STGR	-0.1				
Plant aspect	-0.26**	0.59**			
Ears per plant	-0.82**	-0.24	-0.01		
Ear aspect	0.44**	-0.19	-0.01	-0.49**	
Grain yield	-0.43**	0.15	-0.26**	0.29**	-0.31**
High N environment					
STGR	0.20**				
Plant aspect	0.28**	0.54**			
Ears per plant	-0.67**	-0.50**	-0.66**		
Ear aspect	0.09	0.52**	0.50**	0.24**	
Grain yield	-0.08	-0.41**	-0.56**	0.32**	-0.26**

STGR, stay-green characteristic. **(p ≤ 0.01)

### 3.2 Summary statistics for SNP markers

Among the 1,660 SNPs, the base pair changes were A/C (192), A/G (631), A/T (15), C/T (621), T/G (172) and G/C (29). A/G and C/T represented the highest base pair transitions among the SNP, accounting for 38.0% and 37.4%, respectively. The distribution of SNPs per chromosome varied between 117 for chromosome 7 to 334 for chromosome 1. The minor allele frequency of 64.9% of the SNPs used for the study was >0.02. The major allele frequency varied between 0.48 to 0.95 (mean = 0.73); the heterozygosity of the markers varied between 0.00 and 0.19 (mean = 0.07); the polymorphic information (PIC) varied between 0.09 and 0.56 (mean = 0.29).

### 3.3 Genetic relationship and population structure

**Figure 1:**
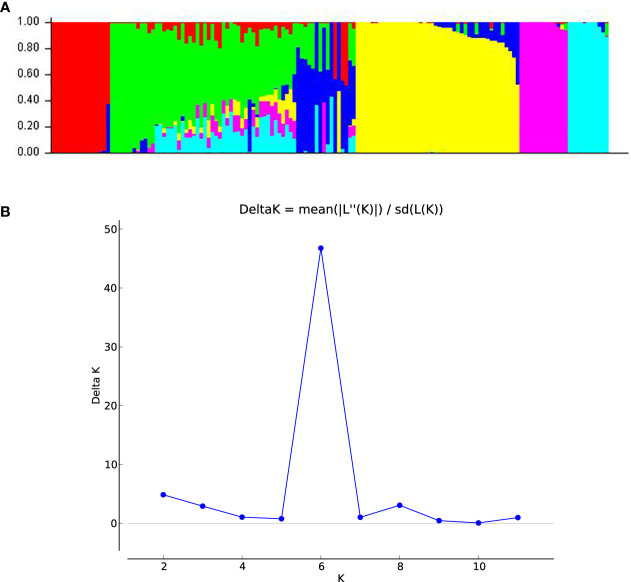
**(A)** A bar plot of population structure analysis revealing six sub-populations based on SNP markers. **(B)** A graph indicating the best K according to the Evanno method based on which the genotypes were classified into sub-populations at k = 6 via SNP markers.

**Figure 2:**
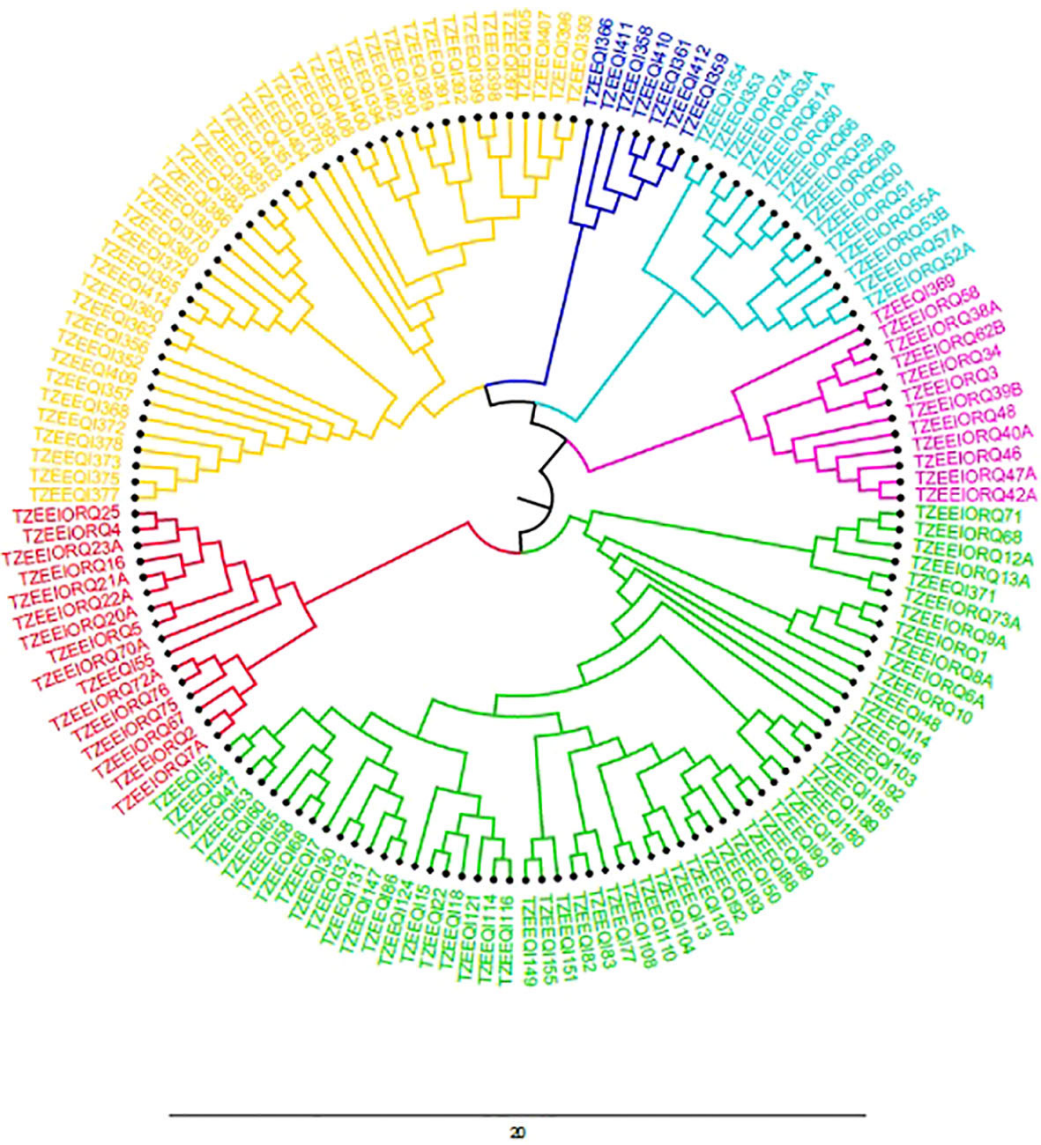
Phylogenetic tree depicting the clustering of the inbred lines into six distinct groups based on SNP markers via the Nei’s method.

The population structure analysis revealed the peak of the *ad hoc* K as K= 6, which indicated that the inbred lines belonged to six distinct populations (**Figure 1b**). Sub-populations 1 to 6 consisted of 15 (10.0%), 20 (13.3%), 2 (1.3%), 43 (28.7%), 13 (8.7%) and 11 (7.3%) genotypes, respectively, which represented 69.33% of the lines. Forty-six (46) genotypes (30.67%) were classified as mixed populations because their probability of association was less than the 70% threshold for any specific population. The classification of the genotypes into sub-populations followed a pattern that could be attributed to the ancestry, pedigree and endosperm colour except for sub-population 3 which had mixed endosperm. Sub-population 1 consisted solely of inbred lines of yellow endosperm; sub-population 2 had 6 orange with 14 genotypes having yellow endosperm lines; sub-population 3 had yellow endosperm only; sub-population 4 had solely white lines; sub-population 5 was composed solely of white genotypes; sub-population 6 was composed of orange lines only. The estimated mean fixation index (F_S_T), which indicated the extent of divergence in each of the six sub-populations were given as 0.87, 0.43, 0.54, 0.63, 0.77 and 0.74 for populations 1 to 6 respectively. The unrooted neighbor-joining phylogenetic tree displayed six clusters similar to the population structure analysis, although the genotypes that constituted the individual cluster in the NJ tree were different from those in the population structure analysis (**Figure 2**). Cluster 1 consisted of 43 inbred lines that were all from the yellow endosperm sub-population; cluster 2 also had 7 yellow endosperm lines; cluster 3 had 15 (2 yellow and 13 orange endosperm lines); cluster 4 had 12 (1 yellow and 11 orange); cluster 5 had 58 (10 orange, 47 white and 1 yellow); and cluster 6 had 16 (1 yellow and 15 orange). In comparing the clustering pattern in the structure analysis and the phylogenetic tree to the performance under LN and HN, no specific patterns were detected.

### 3.4 Association mapping

**Figure 3:**
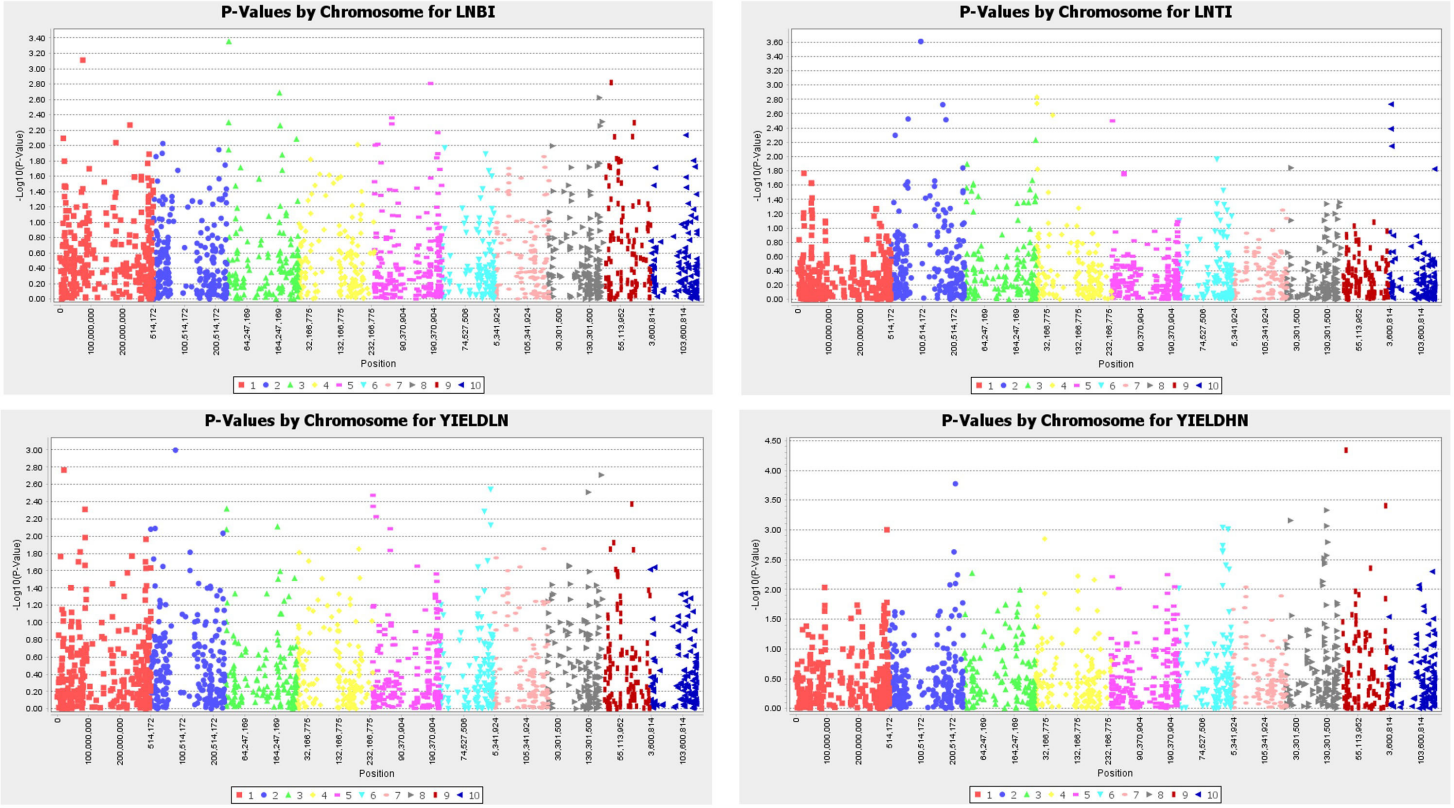
Manhattan plot of key traits under low and high nitrogen conditions; LNBI, low nitrogen base index; LNTI, low nitrogen tolerance index; YIELDLN, grain yield under low nitrogen; YIELDHN, grain yield under low nitrogen.

Fifteen SNPs that reached a threshold of –log10 (p-value) = 3 were identified for the traits studied (**Figure 3**). Under LN, two SNPs each were found to have a significant association with LNBI and LNTI, respectively, while a single SNP had significant association with GY in the LN environments. In contrast, ten SNPS were found to have significant association with GY under the HN conditions. Two out of the 15 SNPs were located on chromosome 1, three were located on chromosome 2, one on chromosome 3 and three each on chromosomes 6, 8 and 9 respectively. However, no coincidental markers were detected for the traits. The significant SNPs had minor allele frequencies ranging from 10% to 50%.

### 3.5 Candidate gene identification and functional annotation

Comparing the positions of the 15 significant SNPs to the B73 reference genome version 3 enabled identification of candidate genes for all the traits. Majority of the SNPs co-localized their candidate genes, as they were mapped within the genes, with few exceptions. *GRMZM2G380319* and *GRMZM2G026137* for LNBI on chromosomes 1 and 3 were adjacent to their respective SNPs within 500 bp and 1 kbp sliding window, respectively. Candidate genes *GRMZM2G442057* and *GRMZM2G338056* for grain yield under HN were located within 500 bp sliding window. Interestingly, the two SNPs identified for LNTI on Chromosome 2 were mapped within the same gene *GRMZM2G077863*. Similarly, two gene host spots, *GRMZM2G080314* on chromosome 6 and *GRMZM2G090928* on chromosome 8, were identified for GY under HN as they both harbored two significant SNPs each (**Table 3**). It was observed that all the 15 significant SNPs detected were located within QTL genomic regions previously reported. None of the random SNPs selected for this study was found to be significantly associated with any trait. It was also striking that all the two SNPs (*PZE-101084671* and *PZE-103012466*) significantly associated with LNBI had exact names and position as those reported by previous QTLs studies by Ju et al. (2018). Functional annotations and the putative protein family were retrieved for all the candidate genes except GRMZM2G026137 for LNBI and *GRMZM2G111809* for grain yield in HN environments (**Table 3**).

**Table 3:** List of significant SNP markers linked to low nitrogen base index (LNBI), low nitrogen tolerance index (LNTI), and grain yield under low nitrogen (LN) and high nitrogen (HN) conditions via association mapping.

Trait	ChrNo	SNP ID	Base Transition	Position	P-value	Minor Allele Frequency	Candidate Gene	Gene annotation
LNBI	1	*PZE-101084671*	A/G	73357546	7.72E-04	0.13	*GRMZM2G026137*	–
LNBI	3	*PZE-103012466*	T/C	6650941	4.42E-04	0.36	*GRMZM2G380319*	Putative MCB2 protein
LNTI	2	*PZE-102090548*	C/T	96081718	2.46E-04	0.12	*GRMZM2G077863*	GDSL-like lipase/acylhydrolase putative expressed
LNTI	2	*PZE-102090550*	C/T	96086002	2.46E-04	0.12	*GRMZM2G077863*	GDSL-like lipase/acylhydrolase putative expressed
Yield (LN)	2	*PZE-102086253*	C/T	79050153	1.01E-03	0.34	*AC209972.4_FG005*	Protein kinase
Yield (HN)	1	*SYN27559*	T/C	291864105	9.95E-04	0.26	*GRMZM2G004459*	ATP/GTP/Ca++ binding protein
Yield (HN)	2	*SYN259*	T/C	211523915	1.68E-04	0.39	*GRMZM2G111809*	–
Yield (HN)	6	*PZE-106083594*	G/T	141063673	9.11E-04	0.45	*GRMZM2G442057*	Tetratricopeptide repeat (TPR)-like superfamily protein
Yield (HN)	6	*SYN38086*	G/A	158286621	9.73E-04	0.48	*GRMZM2G080314*	ATBARD1/BARD1 putative expressed
Yield (HN)	6	*SYN38080*	T/C	158287119	9.73E-04	0.48	*GRMZM2G080314*	ATBARD1/BARD1 putative expressed
Yield (HN)	8	*PZE-108014231*	C/T	14071576	6.96E-04	0.33	*GRMZM2G011213*	Mitochondrial glycoprotein family protein
Yield (HN)	8	*PZA01049-1*	A/G	129940935	8.60E-04	0.33	*GRMZM2G090928*	Transmembrane uncharacterized protein
Yield (HN)	8	*PZE-108074836*	T/C	129941989	4.69E-04	0.35	*GRMZM2G090928*	Transmembrane uncharacterized protein
Yield (HN)	9	*PZE-109016384*	G/A	16485622	4.57E-05	0.17	*GRMZM2G338056*	ZOS11-10 - C2H2 zinc finger protein expressed
Yield (HN)	9	*SYN25338*	T/C	143003020	3.93E-04	0.21	*GRMZM2G150598*	ATP-dependent Clp protease ATP-binding protein

ChrNo, chromosome number; LNBI, low nitrogen tolerance base index; LNTI, low nitrogen index.

## 4 Discussion

The variances for genotype were significant in both LN and HN environments for GY and most traits, which indicated the presence of genetic variability among the inbred lines and represented a good genetic basis for identifying genomic regions related to the traits. The study reported high heritability for GY under HN, which suggests that the phenotypic variations observed among the inbred lines were largely due to the effects of genes on the trait relative to the effects of the environment. The heritability of GY in HN conditions were higher compared to LN which indicated that the possibility of identifying relevant genes for high GY in the HN environment was relatively higher compared to the LN environments. This was confirmed by the association mapping study which identified 10 significant SNP markers and nine candidate genes for GY in the HN conditions compared to only one marker and a candidate gene in the LN conditions. Low to moderate heritability estimates of GY under non-optimum conditions have been previously reported by other studies (Bänziger et al., 2000; Badu-Apraku et al., 2004) which justifies the need to identify markers for enhanced genetic gain from selection. The existence of significant genetic variance together with moderate to high heritability for the majority of the traits indicated a high resolution for the identification of SNPs or genomic regions associated with the traits (Morosini et al., 2017; Alves et al., 2019). The significant pairwise genetic correlation observed between GY and other secondary traits indicated that a common genetic base could possibly underline the phenotypic variations observed among the lines. Alves et al. (2019) demonstrated that traits that exhibit strong pairwise genetic correlation are controlled by the same genomic regions with pleiotropic genes or could be due to linkage. It is therefore anticipated that the significant SNPs detected for LNBI could enhance progress in breeding for LN tolerance when used together with the base index in MAS. In this study, higher STGR (delayed senescence) were observed under the HN compared to the LN environments which indicated that chlorophyll retention was lower under N stress. This suggests that plants that demonstrated better STGR had improved N assimilation and probably possess genes for improved N uptake. However, different correlation patterns were observed between GY and STGR under the LN and HN environments. The correlation between GY and STGR was not significant under LN environment, however, a significant and negative correlation was observed under HN environment. This suggests that retaining chlorophyll in the leaves under stress conditions does not guarantee that the plant would remobilize the nitrogen or photosynthate directly for grain filling, hence there was no benefit for grain filling. The negative correlation observed between GY and STGR under HN indicates that delayed senescence extends the period of grain filling thereby, enhancing grain yield under HN environments. The observed yield penalty of 44% under LN indicated that the stress imposed was appropriate for the expression of genes for LN tolerance because it was within the threshold yield loss of 10-50% reported by Wolfe et al. (1988).

Understanding the genetic variation between and within populations is critical for maximizing the potential of germplasm in breeding programmes and for increasing the selection efficiency for variety development (Makumbi et al., 2011). In this study, the grouping of the inbred lines by both the admixture population structure and cluster analysis into the six sub-population was largely consistent with the pedigree, ancestry and the endosperm colour of the three populations. Although the majority of the markers used were located within or near genomic regions responsible for LN-related traits hence were expected to discriminate the lines according to their performance under LN, no such pattern was detected. The inability of the lines to be grouped according to their performance under low soil nitrogen conditions conformed with the results of Xia et al. (2004) and Warburton et al. (2002) who found that inbred lines from varying populations are more likely to cluster according to their genetic distances rather than the environmental performance.

The association mapping analysis identified a set of 15 SNPs for the four traits studied; ten SNPs were detected for GY under HN. The higher number of markers associations for GY under HN relative to GY under LN could be attributed to the high environmental influence and lower heritability of GY under LN. The absence of significant coincidental marker associations among the studied traits was surprising, especially for LNBI and LNTI, which are both methods of estimating the level of LN tolerance. This suggested that several genomic regions are responsible for LN is a quantitatively inherited trait. The higher number of significant SNP associations for grain yield under HN and the lack of pleiotropic genetic effects among the traits is corroborated by reports that grain yield and nitrogen use efficiency are complex and quantitatively inherited (Kant et al., 2011; Morosini et al., 2017) and are highly influenced by genotype x environment effects (Gallais and Hirel, 2004; Morosini et al., 2017).

The 15 significant SNPs identified were positioned within QTLs previously identified for LN associated traits (Almeida et al., 2013; 2014; Trachsel et al., 2016; Bouchet et al., 2017; Mandolino et al., 2018; Morosini et al., 2017; Ribeiro et al., 2018; Ju et al., 2018), none of the random SNPs selected were significantly associated with the traits. Furthermore, 11 out of the 15 SNPs were mapped within genes. This suggested that the identified SNPs could be useful in MAS to integrate these genomic regions into populations to improve their tolerance to LN and increase the grain yield. The two SNPs, *PZE-102090548* and *PZE-102090550*, found to have high significant association with LNTI were mapped to the same candidate gene *GRMZM2G077863*, suggesting a gene hotspot for this trait. This gene encodes a putative protein and is a member of the GDSL esterase/lipase gene family. The proteins in this family have been described as multifunctional proteins involved in several cellular, molecular and biological processes including lipid metabolic processes (Chepyshko et al., 2012). This gene has been found to exhibit a high expression pattern in the roots of young seedlings at approximately 6 DAP and during tassel meiosis just before flowering (Andorf et al., 2010). Similarly, two SNPs, *SYN38086* and *SYN38080*, which had high significant association with grain yield under high nitrogen condition were mapped to the gene *GRMZM2G080314*. The gene is a ATBARD1/BARD1 protein involved in DNA repair in plants (Reidt et al., 2006). This gene exhibits high expression patterns in the immature tassel at the vegetative stage and in the immature cob at flowering (Andorf et al., 2010). The two SNPs, *PZA01049–1* and *PZE-108074836*, with high significant association with were mapped to the same gene *GRMZM2G090928.* This gene is a putative transmembrane protein that is known to transport specific substances across cell membranes and is also involved in plant cell proliferation (Chen et al., 2003). The gene exhibited a high expression pattern in anthers during the silking stage at flowering (Andorf et al., 2010). The candidate gene *AC209972.4_FG005*, which is associated with grain yield under LN, belongs to the putative protein kinases family which are known to regulate the expression of other genes. They act as switches in cells by phosphorylating target proteins for expression (McClendon et al., 2014). This gene has been found to exhibit a high expression pattern in the primary roots of young maize seedlings around 9 DAP, in the leaves at the five-leaf stage, and during the yield formation period (Sekhon et al., 2011; Andorf et al., 2010). For the genes associated with LNBI, functional annotation was not found for *GRMZM2G026137*, and no information was available on the gene ontology. However, the gene has been reported to be highly expressed in the anthers at the silking stage of the maize plant (Andorf et al., 2010). The other putative gene, *GRMZM2G380319*, was discovered to be significantly associated with LNBI and is an MCB2 protein that has been previously associated with transcription regulatory functions (Hunt et al., 2018). However, the expression of this gene has not yet been linked to any specific plant part. The candidate gene *GRMZM2G00445*9, associated with grain yield under HN, is a putative ATP/GTP/Ca++ binding protein, which is a regulatory protein involved in several molecular and biological functions. This protein was previously reported to play a major role in several biological processes, including cell growth, reproduction and post-embryonic development (Goodstein et al., 2012). High expression of this gene in maize has been observed in the primary root post-emergence and in the immature cob at flowering (Andorf et al., 2010). *GRMZM2G442057*, identified as a candidate gene for grain yield under HN, belongs to the tetratricopeptide repeat (TPR)-like superfamily protein. TPR proteins play a significant role in the regulation of various cellular functions (Rosado et al., 2006). *GRMZM2G011213* is a mitochondrial glycoprotein involved in cellular mitochondrial functions, mainly for cell energy production (Goodstein et al., 2012). The gene demonstrated remarkable expression pattern in the roots of young seedlings at about 6 DAP and in the leaves at the vegetative stage (Andorf et al., 2010). *GRMZM2G338056* is a putative ZOS11-10 - C2H2 zinc finger protein involved in molecular functions such as sequence-specific DNA binding. The gene is substantially expressed in both the primary roots and shoots at the three-leaf stage and in the silks during the silking stage of flowering. *GRMZM2G150598* is a potential ATP-dependent protease ATP-binding protein and protein linked to molecular functions such as protein binding, for biological processes such as protein metabolism and as cellular components of plastids. The gene is highly expressed in the leaves of seedlings at the emergence and three-leaf stage and later in the topmost leaves just before flowering (Andorf et al., 2010). Although functional annotation was not available for *GRMZM2G111809*, data available in the Maize GDB suggested substantial expression patterns of the gene in the roots of young seedlings after emergence and in immature cobs at flowering (Andorf et al., 2010). The candidate genes associated with LNBI and LNTI have been found to be highly expressed in the plant roots possibly for enhanced nitrogen uptake. Morosini et al. (2017) reported higher root length in maize under LN stress compared to HN environment. Higher root length under LN stress is an adaptive response mechanism aimed at conserving plant nitrogen while increasing the potential for nitrogen uptake from the soil (Morosini et al., 2017). It has also been previously reported that a common response of maize plant to soil nitrogen deficiency is an increased root length and altered root architecture (Gaudin et al., 2011). Interestingly, one of the SNPs (*PZE-103012466*) significantly associated with LNBI had been previously reported to flank QTL for root length under LN stress in a study by Ju et al. (2018). This indicated that the candidate gene *GRMZM2G380319* possibly caused an increased root length under LN for maximum nitrogen uptake. The marker *PZE-103012466* together with the other markers found in this research could potentially be employed in MAS to select for increased nitrogen uptake or tolerance to LN among our QPM inbred lines and other tropical and non-tropical maize populations.

## 5 Conclusion

Both the population structure analysis and phylogenetic tree grouped the inbred lines into six distinct sub-populations, primarily based on their ancestral origins, pedigree information, and endosperm colour. Fifteen SNP markers were found to have significant associations with the low nitrogen tolerance base index (LNBI), the low nitrogen tolerance index (LNTI), and grain yield in both high nitrogen and low nitrogen environments. All the significant SNPs were located within QTLs previously reported for LN tolerance and related traits. The majority of these SNPs co-localized genes with a few adjacent to their candidate genes within 500 bp or 1 kbp sliding window. Two SNPs significantly associated with LNTI were mapped within the same gene, *GRMZM2G077863*, indicating that this gene is strongly associated with LN tolerance. Similarly, *GRMZM2G080314* co-localized two significant SNPs detected for high grain yield under HN conditions. Functional annotations for the candidate gene revealed that a large majority of the candidate genes were involved in root functions, expressed for tassel meiosis prior to flowering, or in the anther and silk during flowering. The marker *PZE-103012466* associated with LNBI was found to have the same SNP ID and position as a SNP marker previously reported for increased root length for nitrogen uptake; hence, it could be used for MAS in our population and other tropical populations. The 15 identified markers could be useful to breeding programmes for LN tolerance breeding through MAS because they are located within known QTLs and were mapped within or very close to genes related to the traits.

## Data Availability

The raw data supporting the conclusions of this article will be made available by the authors, without undue reservation.
